# Supplementary material for: Do Primary Health Professionals in Brazil Have a Misperception about Food? The Role of Food Literacy as a Positive Predictor
Source: Nutrients. 2024 Jan 19;16(2):302. doi: 10.3390/nu16020302 (PMC10821131; doi:10.3390/nu16020302)
Supplement: Supplementary file 1 [file nutrients-16-00302-s001.zip › nutrients-2799637-supplementary.pdf]

Table – Determined categories, meaning cores of interviews, number of occurrences and number of participants.

Category	Meaning core	Occurrence (number of quotes)	Number of participants	
			Professionals	Users
1. Why corn chips are unhealthy?				
Poor on nutrients	Excess of fat	4	4	0
	Excess of salt	17	4	13
	Excess of sugar	1	1	0
	Low on micronutrients	4	3	1
Processing degree	Use of conservatives	13	6	7
	Use of artificial food dyes	9	4	5
	Ultraprocessed or industrialized foods	12	8	4
2. Why fruit in syrup are unhealthy?				
Poor on nutrients	Excess of salt	1	0	1
	Excess of sugar	21	17	4
Processing degree	Use of conservatives	14	7	7
	Ultraprocessed or industrialized foods	5	2	3
3. Why stuffed cookies are unhealthy?				
Nutrients	Excess of fat	15	8	7
	Excess of salt	6	4	2
	Excess of sugar	17	11	6
	Low on nutrients	3	2	1
Processing degree	Use of conservatives	6	4	2
	Use of artificial food dyes	8	6	2
	Ultraprocessed or industrialized foods	7	6	1
	Due to the cookie filling	9	5	4
**4. Do you believe that cream crackers are good or bad for your health?**				
Usability	Used in hospital diets	3	1	2
	Health professionals (physicians and nutritionist) indicate	3	1	2
	Affordable source of carbohydrates	1	1	0
Rich on nutrients	Good composition (it is only water and salt)	5	1	4
Poor on nutirents	Excess of fat	1	1	0
	Excess of salt	4	4	0
Processing degree	Use of conservatives	4	4	0
	Ultraprocessed or industrialized foods	8	7	1
Neutral	I do not know	4	2	2
	It is not good nor bad	3	1	2
	Better than stuffed cookie	11	9	2
	It depends the consumed amount	5	3	2
**5. Do you believe that ham is good or bad for your health?**				
Poor on nutirents	Excess of fat	3	1	2
	Excess of salt	6	4	2
	Use of conservatives	10	3	7
Processing degree	Ultraprocessed or industrialized foods	14	12	2

	Processed meat	10	8	2
Neutral	I do not know	6	1	5
	It depends the consumed amount	3	3	0
**6. Do you believe that soybean oil is good or bad for your health?**				
Rich on nutrients	Because it is of plant origin	2	2	0
Poor on nutrients	Excess of fat	6	3	3
	Worse than other oils	15	8	7
	It is bad when used for frying	5	5	0
	Raises cholesterol levels	5	3	2
	I couldn't explain why (but it is bad)	5	1	4
Neutral	I do not know	5	5	0
	It is cheap	5	4	1
	It depends the consumed amount	7	3	4
**7. Do you believe that light foods are good or bad for your health?**				
Rich on nutrients	Contains less fat	4	1	3
	Decrease in some nutrients	3	2	1
Usability	Used in specific situations (diabetics, high cholesterol levels, special diets, athletes)	5	3	2
	They do “less harm” than common foods	3	2	1
	I couldn't explain why (but it is good)	2	0	2
Processing degree	Ultraprocessed or industrialized foods	4	3	1
Neutral	I do not know	6	4	2
	I do not know what light food is	3	2	1
	It is not good nor bad	1	0	1
	It depends the consumed amount	4	3	1
**8. Why do you believe that processed turkey breast is good for your health?**				
Rich on nutrients	Less fat/calories	6	3	3
	Less preservatives	1	0	1
	It is cooked (not fryed)	1	0	1
	Source of fiber	1	0	1
Poor on nutrients	Excess of salt	4	3	1
	It is a processed meat	8	7	1
	Use of conservatives	5	4	1
Processing degree	Ultraprocessed or industrialized foods	8	6	2
Neutral	I didn't know how to answer	8	4	4
Usability	Best option within sausages and processed meat	9	6	3
**9. Why do you believe that gelatin is good for your health?**				
Usability	Suitable for diets/patients	7	5	2
	Suitable for pregnant women	1	1	0
	Suitable for the elderly	1	1	0
	Recommended by doctors	3	1	2
	Good for hydration	3	2	1
Rich on nutrients	Rich in calcium	2	1	1
	Rich in collagen	17	11	6
	It is a soft food	3	2	1
Poor on nutrients	Excess of sugar	6	6	0
Processing degree	Use of artificial food dyes	7	6	1
	Ultraprocessed or industrialized foods	2	1	1
Neutral	I do not know	2	0	2
	It depends the consumed amount	1	1	0

**10. Why do you believe that coconut oil is good for your health?**				
Rich on nutrients	Less preservatives	2	2	0
	Natural (fruit source)	15	7	8
	Less fat	2	2	0
	I don't know why (but is good)	2	0	2
Usability	Better than Other oils	11	9	2
	Reccomended in the media	3	1	2
Poor on nutrients	Raises LDL and triglyceride levels	2	2	0
Neutral	I do not know	5	3	2
	It depends the consumed amount	3	3	0
**11. Why do you believe that honey is good for your health?**				
Usability	Good for flu	4	1	3
	Has anti-inflammatory action	5	5	0
	Natural food	17	9	8
Rich on nutrients	Has antioxidants	1	1	0
	I don't know why (but is good)	1	1	0
	Better than sugar	5	5	0
Poor on nutrients	Excess of sugar	5	4	1
Neutral	I do not know	2	0	2
	It depends the consumed amount	4	4	0
	It depends from the source	10	6	4
